# Locked Wire Fixator for a Distal Radius Fracture With Malunion: A Case Report

**DOI:** 10.7759/cureus.50193

**Published:** 2023-12-08

**Authors:** Marika Nunotani, Kiyohito Naito, Nana Nagura, So Kawakita, Muneaki Ishijima

**Affiliations:** 1 Department of Orthopaedics, Juntendo University, Tokyo, JPN; 2 Department of Medicine for Orthopaedics and Motor Organ, Juntendo University Graduate School of Medicine, Tokyo, JPN

**Keywords:** existing volar locking plates, locked wire fixator, residual convex deformity, malunion, distal radius fracture

## Abstract

Surgical treatment for a distal radius fracture using a volar locking plate is difficult if the distal radius malunion remains. Therefore, a different surgical method from volar locking plate fixation should be required. We report the case of an 83-year-old woman with a left dorsal displaced distal radius fracture. However, the deformity of the volar cortex of the radius was recognized because of a previous distal radius fracture. Therefore, osteosynthesis with a locked wire fixator was performed. At 12 months after surgery, the patient has returned to daily activities without difficulty. A locked wire fixator can be useful for treating a distal radius fracture with malunion of the volar cortex of the distal radius.

## Introduction

Currently, about 60% of distal radius fractures are treated conservatively, although surgical treatment is increasing [1]. Malunion occurs in approximately 23% of cases when conservative treatment is chosen for distal radius fractures [2]. Deviation from the normal range of volar tilt can lead to a limited range of motion of the wrist and forearm, decreased grip strength, and ulnar-sided wrist pain due to ulnar head-lunate impingement caused by radial shortening [3]. In addition, these complications caused by malunion are known to lead to decreased activities of daily life (ADLs) [3].

When symptoms of malunion interfere with ADLs such as pain, limitation of range of motion, and decreased grip strength, corrective osteotomy should be considered. A wedge osteotomy of the malunion and fixation with a volar locking plate is widely used to correct malalignment of a radius [4]. However, if the existing volar locking plates do not conform to the shape of the distal radius after osteotomy, there is a risk of volar soft tissue injuries, including flexor tendon injuries, due to the volar locking plate. In such cases, surgeons need to devise ways to prevent these complications [5-7].

In this report, the authors present a case where a malunited distal radius fracture was refractured. Considering the difficulties of the volar locking plate in the treatment for malunion of the distal radius in this case, osteosynthesis with a locked wire fixator was performed, which resulted in a favorable postoperative outcome.

## Case presentation

**Figure 1:**
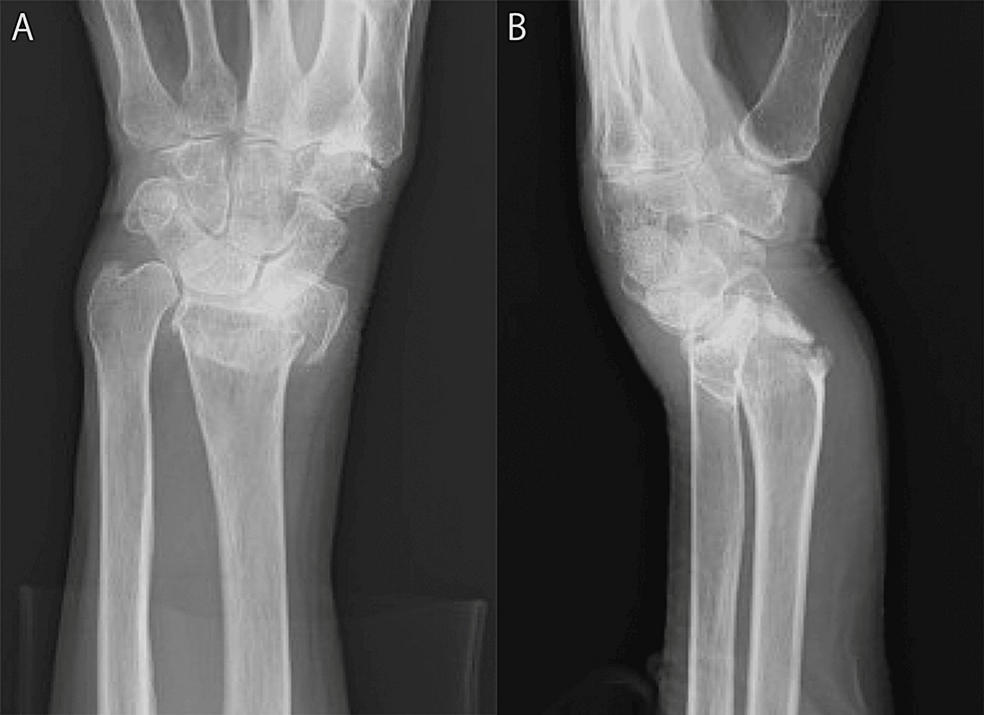
Distal radius fracture with malunion of the volar cortex of the distal radius. Frontal view (A) and lateral view (B) on plain radiography.

**Figure 2:**
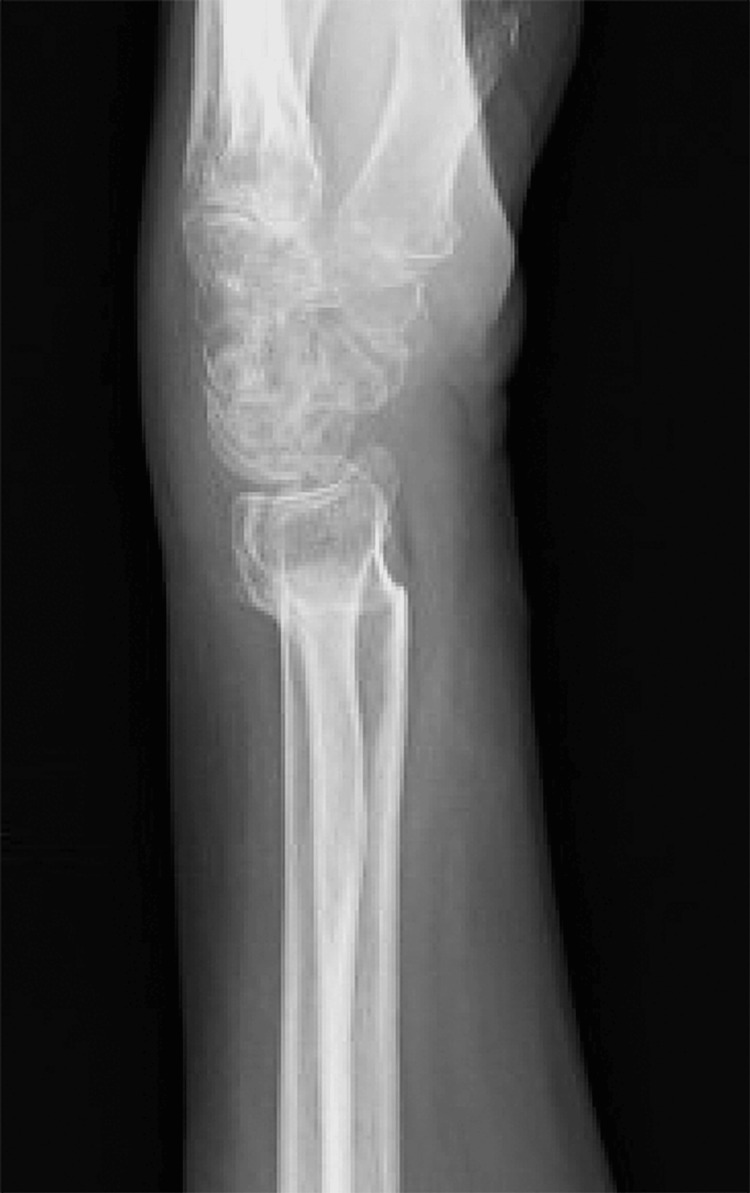
Lateral view on plain radiography 12 years earlier. Lateral view on plain radiography 12 years earlier showed a convex deformity of the volar cortex of the distal radius and a dorsal displacement (volar tilt: -10°).

The patient was an 83-year-old woman with a left dorsal displaced distal radius fracture caused by falling. On plain radiography, shortened radius and severe dorsal displacement (AO classification type C1, volar tilt: -53°, radial inclination: 3°, ulnar variance: 8.7 mm) were noted at the time of injury (Figures 1A, 1B). Closed reduction was performed immediately, but the dorsal displacement was not reduced (volar tilt: -50°). A lateral view on plain radiography showed a convex deformity of the volar cortex of the distal radius (Figure 1B). Twelve years earlier, the patient had received conservative treatment for a distal radius fracture on the same side, which showed malunion of the radius: a convex deformity of the volar cortex of the distal radius and a dorsal displacement (volar tilt: -10°, radial inclination: 10°, ulnar variance: 0 mm) (Figure 2). In this case, surgical treatment was considered for three reasons. The first is the intra-articular fracture line was on the load axis of the lunate in the condition of dorsal displacement, and the second is there was concern that volar tilt (-50°) obtained by closed reduction may lead to the displacement of intra-articular fractures. Furthermore, although the patient required support from the family who were living together, ADLs are maintaining independent living.

**Figure 3:**
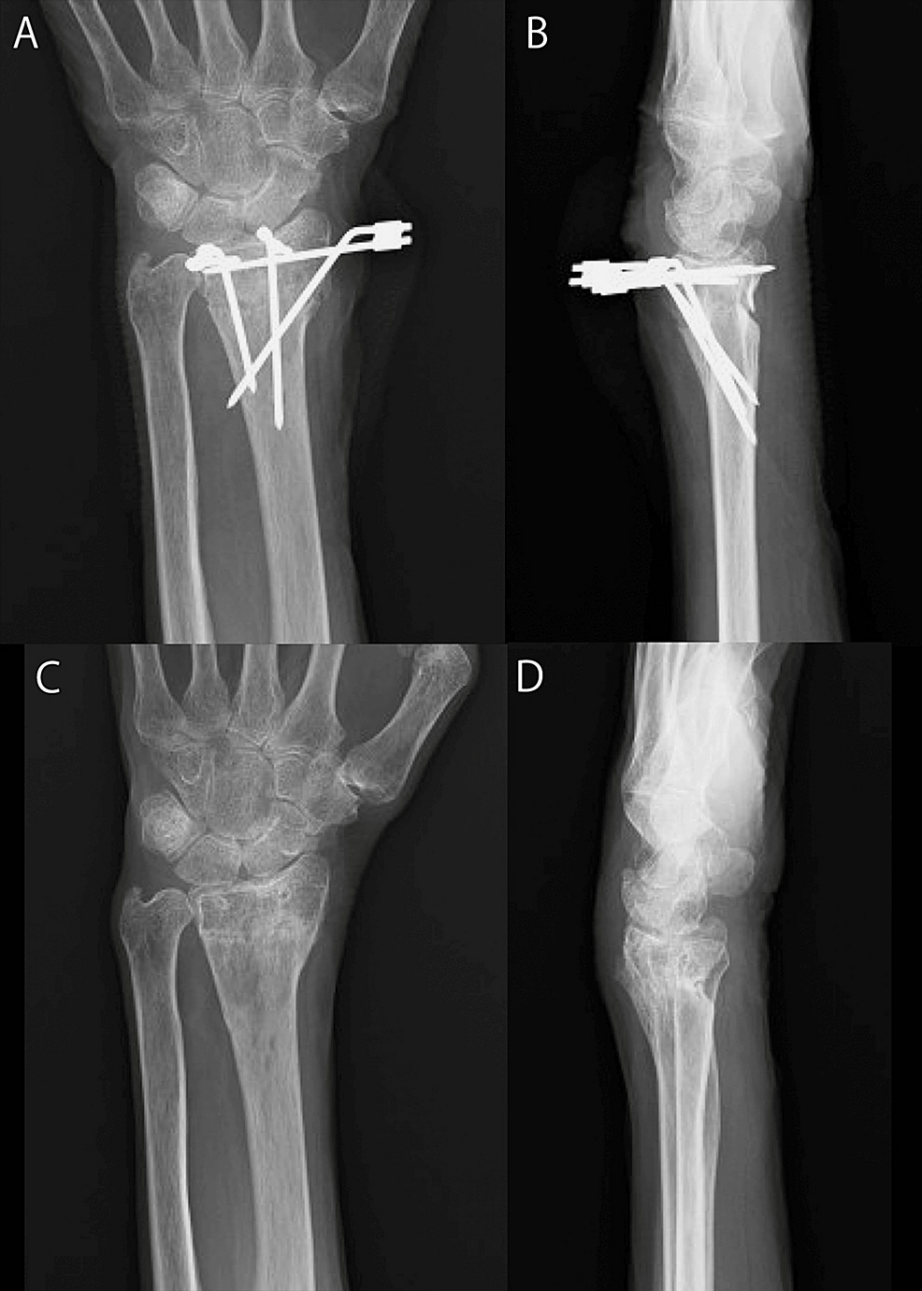
Plain radiography after osteosynthesis with a locked wire fixator. Frontal view (A) and lateral view (B) after osteosynthesis. Frontal view (C) and lateral view (D) 12 months after osteosynthesis.

As the volar cortex of the distal radius was irregular (Figure 2) and the shape of the normal volar locking plate could not be matched to the irregular cortex, osteosynthesis with a locked wire fixator was performed. Surgery was performed in the supine position under a brachial plexus block. First, to reduce the volar tilt, intrafocal pin technique from the dorsal side was used by Kirschner wire. Second, to reduce the radial inclination, intrafocal pin technique from the radial side was used by Kirschner wire. After confirming that volar tilt and radial inclination were reduced, two 1.8-mm mini-fixator pins were inserted from the dorsal side and one 1.8-mm mini-fixator pin was inserted from the radial side (Mini-fixator pin, Arata Co., Ltd., Tokyo, Japan). In addition, two 1.8-mm mini-fixator pins were inserted from the dorsal side and the other was inserted from the radial side into the distal bone fragment. All intrafocal pin techniques and pin insertions were performed percutaneously. Finally, three pairs of 1.8-mm mini-fixator pins, two dorsal and one radial, were connected using a locked wire fixator (JuNctionR, Arata Co., Ltd., Tokyo, Japan) (Figure 3, Panels A and B).

No postoperative immobilization was needed, and the movement of the wrist and fingers was permitted soon after surgery. Six weeks after surgery, the locked wire fixator was removed due to the presence of a callus on plain radiography. Twelve months after surgery, the range of motion of the wrist was flexion, 45°; extension, 45°; forearm pronation, 80°; forearm supination 80°, indicating a mild limitation of wrist range of motion. Bone union was noted on plain radiography (volar tilt: 0°, radial inclination: 13°, ulnar variance: 0 mm) (Figure 3, Panels C and D). However, the Visual Analog Scale score was 3/10, the Q-DASH score was 2.27/100, and the Mayo wrist score was 90/100, which was excellent. The patient returned to ADLs. Furthermore, there were no complications from the locked wire fixator.

## Discussion

There is discussion regarding the extent to which malunion of distal radius fracture is acceptable. Some studies have suggested that the parameters on plain radiography, such as volar tilt, ulnar variance, and intra-articular, gap should be indications for corrective osteotomy [8,9]. On the other hand, Evans et al. suggested that corrective osteotomy should be performed in cases of limited range of motion or pain that interferes with ADLs, regardless of the alignment of the radius or wrist [6]. Although the indications for surgery remain controversial, the basic idea was the same “Alignment that interferes with ADLs should not be tolerated.”

In this case, surgical treatment was considered for two reasons. The first is the intra-articular fracture line was on the load axis of the lunate in the condition of volar displacement, and the second is there was concern that volar tilt with closed reduction may lead to the displacement of intra-articular fractures. In normal anatomy, it has been reported that when the upper extremity is loaded with the wrist dorsiflexed, the load can be applied to the dorsal aspect of the lunate and the articular surface slightly volar to the center of the radial lunate fossa [10]. However, in this case, the dorsal aspect of the lunate, which is considered the loading surface, was relative to the dorsal aspect of the radial lunate fossa in the volar tilt -50° alignment. As the intra-articular fracture on the dorsal aspect of the radius was observed, the goal of surgical treatment was to correct the volar tilt and shift the loading surface of the radius to the volar side.

**Figure 4:**
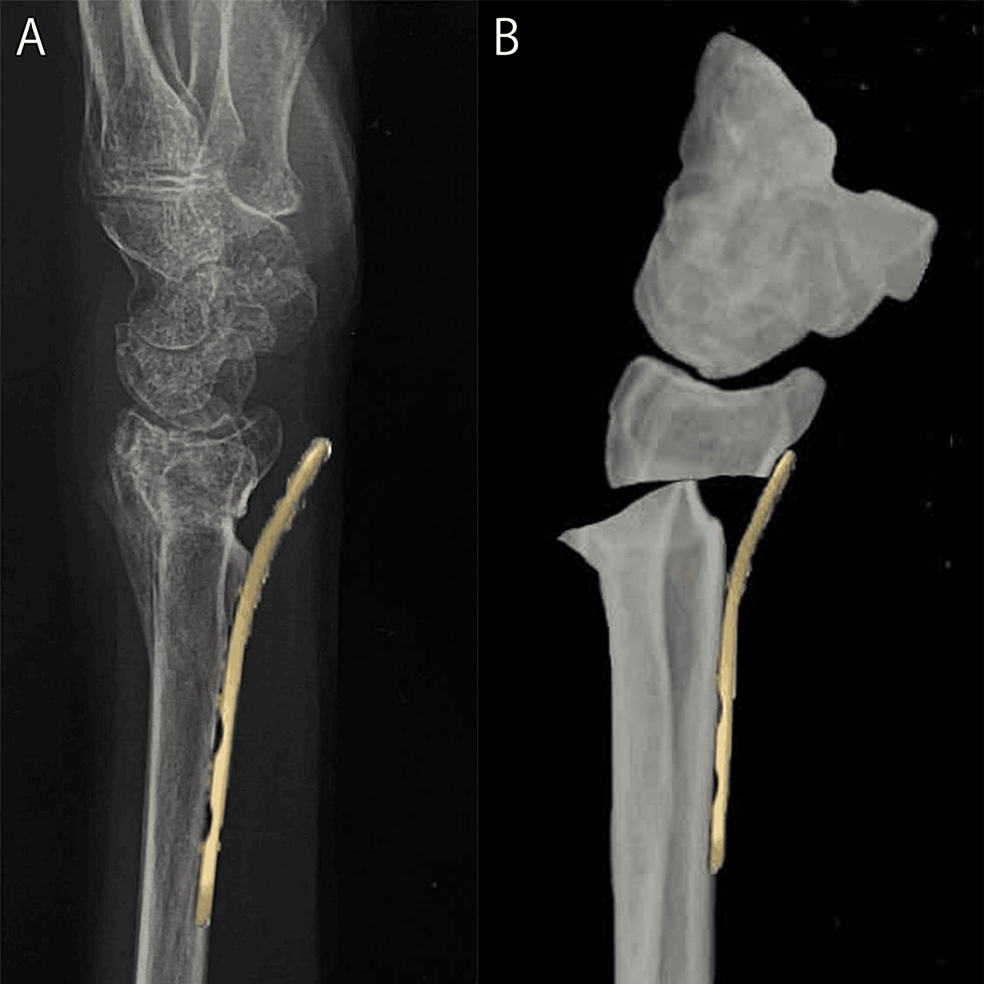
Problems of volar locking plate fixation. A: When the plate is placed on the radial shaft, a gap is caused between the plate and the bone. B: When the plate is placed on the distal fragment, the carpal bones shift volar.

Recently, volar locking plate fixation has become the standard surgical treatment for distal radius fractures [11]. Therefore, we considered the possibility of volar locking plate fixation for this case using two-dimensional images such as plain radiography and CT images. First, when the plate is glued to the radial shaft, a gap is caused between the distal end of the plate and the distal bone fragment (Figure 4A). A floating distal end of the plate is known to increase the risk of flexor tendon injury [12], making such plate placement inappropriate. Second, when the plate is placed in close contact with the distal bone fragment, the carpal bones shift volar from the radial axis (Figure 4B). Such plate placement is also inappropriate because the volar deviation of the carpal bones leads to a limited range of motion of the wrist joint [13]. Moreover, it is difficult to place an existing plate with residual convex deformity.

**Figure 5:**
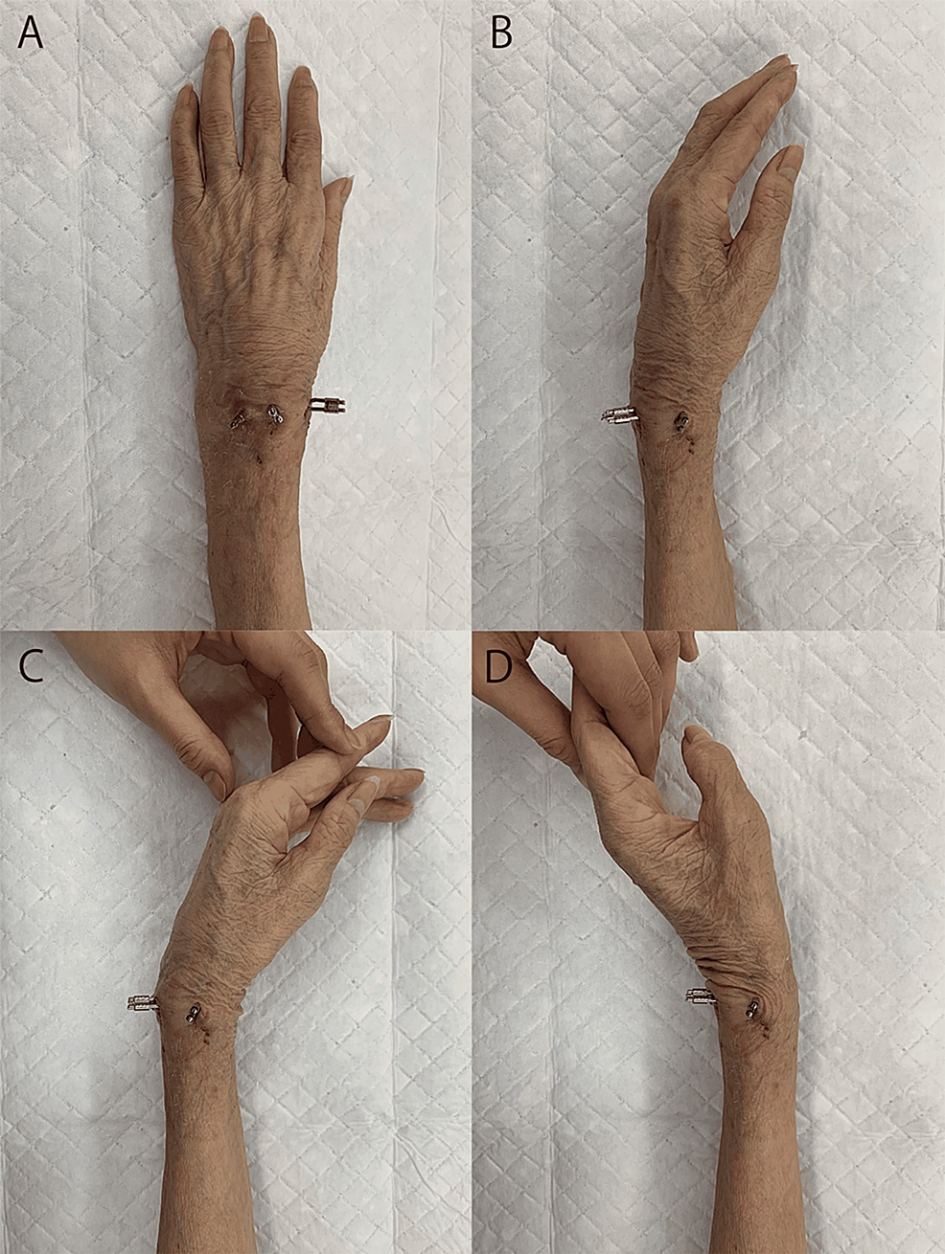
Stabilization by a locked wire fixator. Dorsal view (A) and radial view (B) of the wrist with a locked wire fixator. Wrist range of motion training is possible (C: Flexion, D: Extension).

Therefore, a locked wire fixator that can be expected to provide sufficient fixation regardless of residual deformity was used. Wrist range of motion training is possible even with a locked wire fixator (Figures 5A-5D). A locked wire fixator is an external fixation device that connects wires that exit the body during percutaneous pinning, and was developed to solve the problems of conventional pinning with insufficient fixation and pull-out strength [14]. In this case, two dorsal and one radial intrafocal pins were connected to wires inserted parallel to the articular surface of the corresponding distal bone fragments. As a locked wire fixator can achieve sufficient fixation, the patient did not require postoperative immobilization, and movement of the wrist and fingers was permitted soon after surgery, which may have contributed to the favorable postoperative outcome [14]. Furthermore, the wire insertion used in the locked wire fixator is a viable technique with or without malunion. In other words, unlike the volar locking plate, it is a highly flexible implant that does not depend on the shape of the bone. On the other hand, the disadvantages of a locked wire fixator include a weaker fixation compared to the volar locking plate, an increased risk of pin-site infection, and an increased risk of nerve damage due to percutaneous intervention [14]. However, in this case, no perioperative complications were observed, and a favorable postoperative outcome was achieved.

## Conclusions

Surgical treatment for a distal radius fracture with malunion of the volar cortex of the distal radius using a volar locking plate is difficult as the existing volar locking plates do not conform to the shape of the distal radius malunion. Therefore, there is a risk of volar soft tissue injuries, including flexor tendon injuries, due to the volar locking plate. From this perspective, a locked wire fixator can be useful for treating a distal radius fracture with malunion.
